# T-Cell-Based Immunotherapy for Osteosarcoma: Challenges and Opportunities

**DOI:** 10.3389/fimmu.2016.00353

**Published:** 2016-09-14

**Authors:** Zhan Wang, Binghao Li, Yingqing Ren, Zhaoming Ye

**Affiliations:** ^1^Department of Orthopaedics, Centre for Orthopaedic Research, Orthopaedics Research Institute, The Second Affiliated Hospital, Zhejiang University School of Medicine, Hangzhou, China

**Keywords:** osteosarcoma, adoptive T cell transfer, T cell, tumor microenvironment (TMA), combination strategy

## Abstract

Even though combining surgery with chemotherapy has significantly improved the prognosis of osteosarcoma patients, advanced, metastatic, or recurrent osteosarcomas are often non-responsive to chemotherapy, making development of novel efficient therapeutic methods an urgent need. Adoptive immunotherapy has the potential to be a useful non-surgical modality for treatment of osteosarcoma. Recently, alternative strategies, including immunotherapies using naturally occurring or genetically modified T cells, have been found to hold promise in the treatment of hematologic malignancies and solid tumors. In this review, we will discuss possible T-cell-based therapies against osteosarcoma with a special emphasis on combination strategies to improve the effectiveness of adoptive T cell transfer and, thus, to provide a rationale for the clinical development of immunotherapies.

## Introduction

Osteosarcoma (OS) is an aggressive malignancy of bone thought to originate from mesenchymal stem cells (1). It is a rare tumor that predominantly affects children and young adults (2). The most common sites of metastases are lung (>85%) and bone (3). Current treatment for newly diagnosed osteosarcoma includes three main components: preoperative chemotherapy, surgical resection, and postoperative chemotherapy (4). This management strategy has improved the outcome of patients with localized osteosarcoma. However, patients with advanced, metastatic, and recurrent osteosarcomas continue to experience a quite poor prognosis (5). After aggressive treatment with both surgery and chemotherapy, the 5-year survival rate for osteosarcoma patients with localized disease is about 65% (3), whereas it is less than 20% for patients with metastases (6, 7). The use of adjuvant chemotherapy provides no survival advantage for patients with pulmonary metastases (8).

Therefore, novel therapies for osteosarcoma are urgently needed and of great interest in oncology. It is thought that one class of new therapies, involving cellular immunotherapy, is likely to be effective in osteosarcoma (9, 10). For instance, although dendritic cell (DC) vaccination might not induce T-cell response to osteosarcoma (11), it can be combined with antibodies against certain immunoregulatory molecules (e.g., GITR) to enhance antitumor effects in osteosarcoma (12). However, recent evidence reveals that using DC immunotherapy may elicit cytotoxic T cell response in preclinical osteosarcoma models (13). Inoculating bacterial products into unresectable tumors has been found to stimulate patients’ immune response and inhibit tumor growth (14–16). At this time, the pivotal role of the immune system in antitumor responses is widely accepted. T cells play an essential role in mediating potent tumor-specific immune responses, and may provide a rational basis for tumor immunotherapies, such as adoptive cell transfer (ACT), a quite promising option (17). For example, cluster of differentiation 19 (CD19)-chimeric antigen receptor (CAR)-T cell therapy can mediate potent anti-leukemic activity in children and young adults with chemotherapy-resistant acute lymphoblastic leukemia (18). Importantly, robust cancer regressions have been achieved in patients with metastatic melanoma after using T-cell transfer immunotherapy (19). This suggests a possible role and significant efficacy of T cell-mediated treatment of other solid tumors, including osteosarcoma. Indeed, promising results have been reported recently in studies of adoptive T cell therapy in osteosarcoma (20–24). Results from studies of other solid malignancies, such as melanoma, can point to new immunotherapeutic strategies that may improve survival of patients with advanced osteosarcoma, prevent metastases, and reduce relapse rates in patients with resected tumors.

In this article, we briefly review T-cell-based immunotherapies, discuss their challenges, and consider corresponding solutions. Furthermore, we discuss novel therapeutic strategies for treatment of osteosarcoma, based on our current understanding of the adoptive transfer of unmodified or gene-engineered T cells.

## Adoptive T Cell Transfer for Osteosarcoma

Treating patients with cell populations that have been isolated, manipulated, expanded *ex vivo* and reinfused into patients is defined as ACT. Immunologists generally use one form of adoptive immune cell transfer, notably, adoptive T cell transfer (ATCT). In this process, T cells are infused back into a patient after *ex vivo* expansion, and then migrate to the tumor site and mediate an antitumor effect. The fundamental requirements for successful ATCT have become technically feasible in recent years, and ATCT has become a promising option for cancer treatment, because it has several advantages compared with other forms of immunotherapy. T cells with desired specificities and enhanced functionality for potent antitumor responses can be selected and collected *in vitro*, consequently avoiding adverse reactions *in vivo*. In addition, interleukin-2 (IL-2) can promote T lymphocyte growth *ex vivo* without functional loss of effector T cells (25). This, and other advances in cell culture, have made ATCT technically feasible, because it is now possible to generate sufficient quantities of human T cells for subsequent infusion. And most importantly, tumor microenvironments can now be manipulated to make the lesions more susceptible before the administration of ATCT. These manipulations can include blocking mechanisms of immunosuppression (such as eliminating T-regulatory lymphocytes) that represents a unique advantage of ATCT (26, 27). At this time, the two most pressing questions appear to be: (1) Can new T cell sources be developed, to replace autologous cell production and overcome histocompatibility barriers? (2) What is the best method to minimize on-target or off-target toxic effects of ATCT?

Recent reports of excellent efficacy of ATCT for cancer in early clinical trials have led to increased interest in developing T cell therapy (18, 28, 29). In this section, we primarily examine the current landscape of various T-cell-based immunotherapies for cancer, especially for osteosarcoma. We discuss potentially promising antigen targets or immune checkpoints, which may lead to improved modalities for treatment of osteosarcoma.

### Tumor-Infiltrating Lymphocytes

In the complex microenvironment of neoplasms, tumor-infiltrating lymphocytes (TILs) play a crucial role in regulating development and growth of the lesions. One key feature of TILs is their ability to migrate into or infiltrate tumors, while other T cells may not traffic to tumor sites due to deletion of chemokine receptors (30). Moreover, TIL populations comprise a variable ratio of CD4+ and CD8+ T cells (24), and these TILs have stronger antitumor effects than peripheral blood lymphocytes. Additionally, recent evidence suggests that most TILs are directed to non-self-antigens that are only expressed in tumor tissues, instead of known antigens, reducing the risk of autoimmunity from TIL therapy (31).

Many studies indicate that increased TIL density can improve clinical outcome in patients with advanced cancers (32–34), suggesting potent antitumor reaction of TILs. When encountering tumor antigens, these TILs can directly kill tumor cells and release cytokines, such as IFN-γ, IL-2, and TNF, which are known to mediate antitumor immune responses (35, 36). Adoptive transfer of TILs is the earliest known form of efficacious T-cell therapy for solid tumors and has been predominately developed in patients with melanoma (37, 38). Furthermore, combining TIL transfer with lymphodepleting chemotherapy and radiation has achieved impressive clinical outcomes in patients with metastatic melanoma, and has expanded the use of experimental TIL therapy to patients with other types of cancer (19, 39). Isolating and expanding TILs *ex vivo* from patients with osteosarcoma is not an established clinical technique at present, and the presence of TILs in sarcomas positively correlates with a good prognosis (40–42). This suggests that TIL therapy may have potential as an effective treatment of osteosarcoma. In any case, there are no clinical reports of use of ATCT with TILs for osteosarcoma yet, because at this time, isolation and expansion of TILs from osteosarcoma tissues is unreliable. However, recent advances in genetic engineering may lead to new strategies that will make this therapeutic approach feasible. Higher levels of PD-L1 expression in tumor cells are found to be positively correlated with TILs in osteosarcoma, whereas PD-1 expression is shown to be correlated with progression of the osteosarcomas (43, 44). Increased TIL density and PD-L1 levels predict better outcome of other cancers (32, 34, 45). Thus, more studies addressing ATCT with TILs are urgently needed to elucidate the biology and improve the treatment of osteosarcoma.

**Figure 1:**
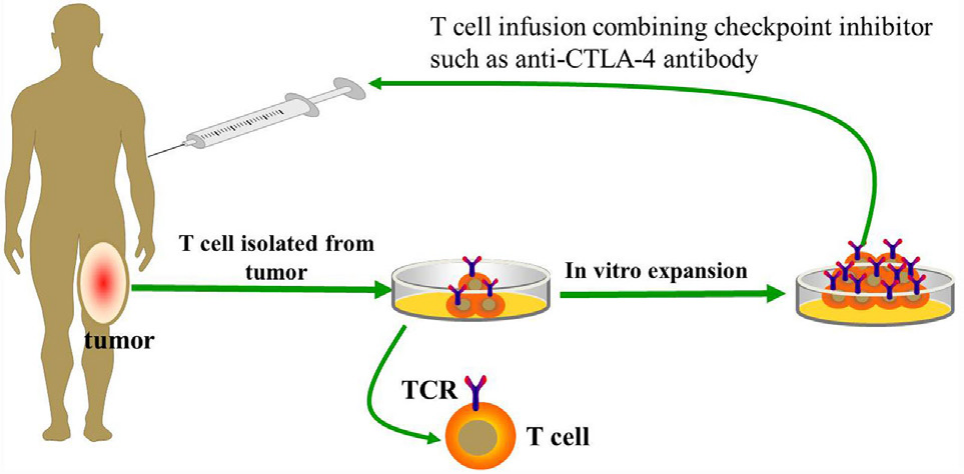
**Basic procedure of adoptive transfer T cells from tumor-infiltrating lymphocytes (TILs)**.

Recently, the first successful isolation of neoantigen-reactive or mutation-reactive T cells from TILs and peripheral blood has been reported, which potentially could lead to development of personalized immunotherapies to treat patients with advanced cancer (46). In the future, the effectiveness of TIL therapy may be further increased, if coupled with the flexible feature of specifically targeting diverse tumor antigens through antigen receptor gene engineering with CARs or T cell receptors (TCRs). Strategies that target mutated tumor-specific antigens (TSAs) are superior to those that target non-mutated self-antigens. The most distinct advantage is that T cells recognizing mutated (“foreign”) antigens are independent of central tolerance and, thus, may potentially express higher-affinity TCR than do those targeting self-antigens (47, 48). However, the frequency of neoantigen-reactive T cells in TIL cultures may potentially limit the effectiveness of this approach. To address this issue, purifying tumor-reactive T cells from bulk TILs and peripheral blood is currently performed via using MHC tetramers generated by candidate neoepitopes identified by whole-exome sequencing (46). Furthermore, recent evidence has shown that neoantigen-specific T cell reactivity can be enhanced by anti-CTLA-4 treatment (47). Therefore, combining checkpoint inhibitors with adoptively transferred neoantigen-specific T cells from TILs or peripheral blood may also represent an effective treatment option for osteosarcoma patients who progress following treatment with individual therapies (Figure 1).

### Unmodified CD8+ T Lymphocytes

**Figure 2:**
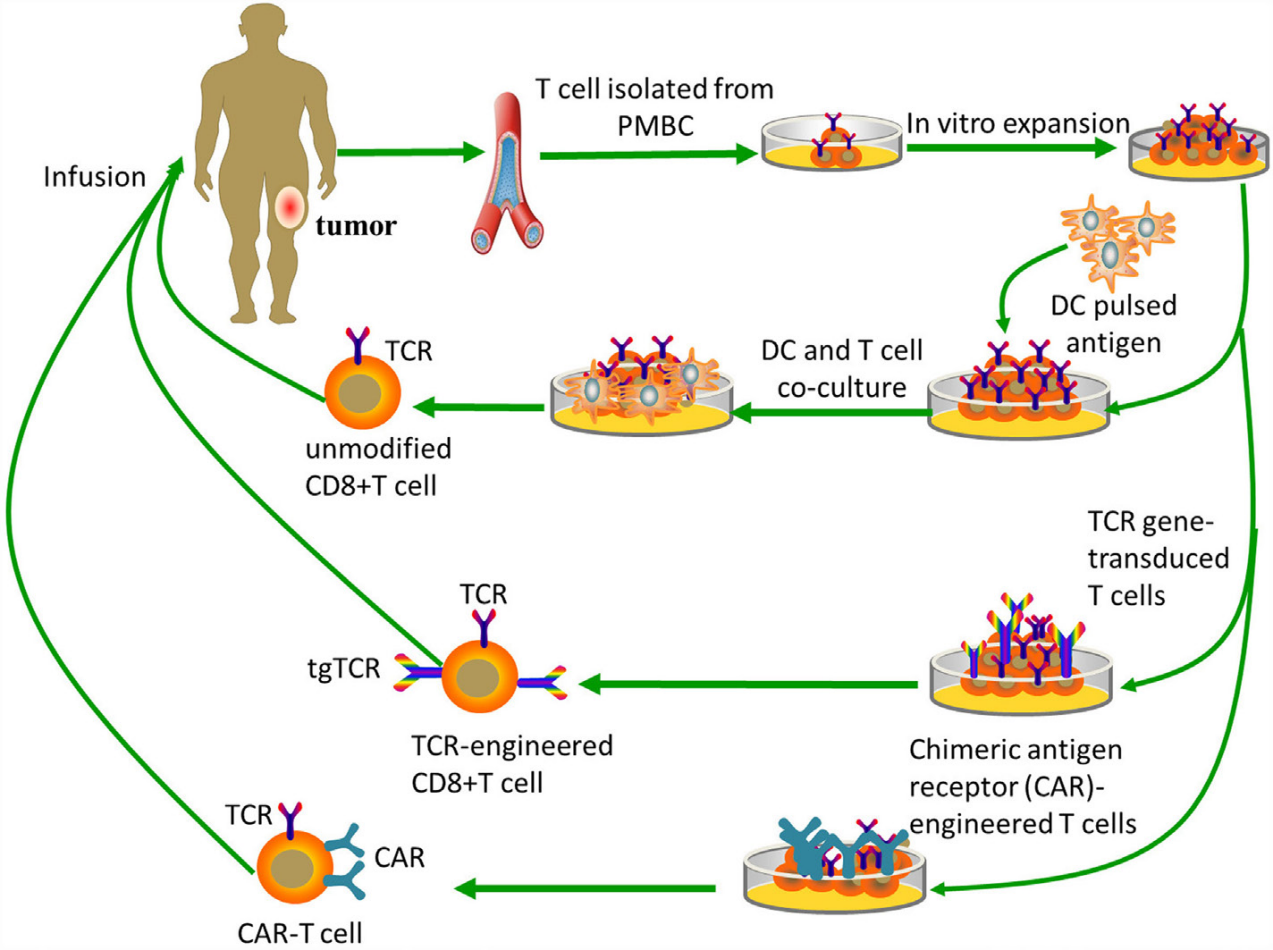
**Tumor-specific T-cell-based immunotherapy**. Unmodified CD8+ T cells are *ex vivo* expanded and do not need genetic modification, while both TCR-engineered CD8+ T cells and CAR-T cells need specific modifications to obtain targeting abilities. Unmodified CD8+ T cells need antigen processing and MHC presentation via antigen-presenting cells, such as dendritic cells. TCR-engineered CD8+ T cells can directly recognize intact target molecules expressed on tumor cell surface in an MHC-dependent fashion, while CAR-T cells are MHC-independent.

Adoptive transfer of tumor-reactive CD8+ cytotoxic T lymphocytes (CTLs) is another promising immunotherapy for treatment of solid tumors (Figure 2). Evidence suggests CTLs have a leading role in immune surveillance of patients with osteosarcoma (49). Finding a TSA that can be reasonably targeted by CD8+ T cells is a key step for the development of adoptive immunotherapy for osteosarcoma. One of the optimal candidates as TSAs for CD8+ T cell recognition is cancer/testis antigen family (CTAs). CTAs are protein antigens, most of which are normally expressed only in human germ line cells, stem cells, and during embryogenesis (50, 51). Due to loss of CTA expression in most normal tissues, these antigens theoretically could elicit immune responses in cancer patients with CTA overexpression. Moreover, CTAs are more often expressed in advanced cancers, indicating that an increased expression of CTAs can be associated with a poor outcome (52). CTAs seem to have important functions in oncogenesis and survival of malignant cells (51). Furthermore, several CTAs, such as the MAGE-A family proteins and LAGE-1/NY-ESO-1, are known to be expressed in osteosarcoma (53, 54). Therefore, CTAs may be promising antigen targets in sarcomas (55, 56).

The specificity of CTAs makes them potential epitopes for antigen-specific adoptive T-cell transfer, and clinical trials using NY-ESO-1 or MAGE-A3 specific lymphocytes against soft-tissue tumors and lung cancer have achieved initial success (57–59). Nevertheless, the level of CTA expression is quite variable among different tumor types. In osteosarcoma, some CTA genes are silenced, complicating the use of CTA-based immunotherapy. However, promising results have been reported in our study evaluating adoptive CD8+ T cell transfer therapy in osteosarcoma. Expression of MAGE-A family and NY-ESO-1 in osteosarcoma cell line U2OS and HOS can be increased following demethylating treatment with decitabine (5-aza-2′-deoxycytidine, DAC). When *in vitro* generated CTA-specific CD8+T cells were reinfused into the osteosarcoma animal models, there was a dramatic antineoplastic reaction and distinct shrinkage of tumors (21). The key for excellent conditions for CTA-specific immunotherapy was increased tumor immunogenicity via elevated CTA expression in the osteosarcoma. Thus, the strategy of using synergistic effects from combining demethylating treatment and specific immunotherapy for control of osteosarcoma should be pursued in clinical trials.

One major drawback of adoptive transfer of CTLs against osteosarcoma is MHC-dependent (limited to patients with certain HLA-haplotypes). Tumor antigens must be presented by HLA to generate effective target recognition (60). Patients with osteosarcoma expressing HLA Class I showed superior overall and event-free survival compared with HLA class I-negative patients. However, deletion or downregulation of HLA class I expression was detected in about half of the osteosarcoma specimens (49). Hence, upregulating HLA, especially HLA class I expression, enhances the antitumor effect of CTLs and can prolong the survival time of patients. Previous studies have revealed that the expression of HLA molecules can be regulated by epigenetic mechanisms (61, 62). Recently, treatment with DAC has also been shown to induce the expression of HLA Class I and/or II molecules in osteosarcoma cell lines (63), further supporting the combination of demethylating treatment and ATCT therapy. When expanding and activating T cells *in vitro*, DCs pulsed tumor lysates or tumor-associated antigens (TAAs) are usually used to co-culture with them. The HLA Class I presenting tumor lysates or TAAs must be compatible with that of CTLs. The design and generation of TAA-specific CTLs should entail HLA compatibility, which is key for the feasibility of clinical applications. HLA expression in common human osteosarcoma cell lines, is summarized in Table 1 (source from www.jaci.jp/HLA.htm).

**Table 1:** **HLAs expression in the common human OS cell lines**.

OS cell line	HLA class I A	HLA class I B	HLA class I C
HOS	0211/–	5201/–	1202/–
U2OS	0201/3201	4402/–	0501/0704
MG63	0101/–	0801/–	0701/–
SaOS-2	0201/2402	1302/4402	0602/0704

Until recently, the adoptive transfer of unmodified CD8+ T cells for osteosarcoma was restricted to preclinical mouse tumor models. Clinical application of T cell therapy for osteosarcoma faces several obstacles before it can be introduced into clinical practice. These include: (1) required production with stringent GMP (good manufacturing practice) procedures in dedicated facilities; (2) necessity of lymphodepleting preparative treatment; and (3) advocate combination of different immunotherapy approaches and integration with conventional treatments (64).

### γδ T Cells

**Figure 3:**
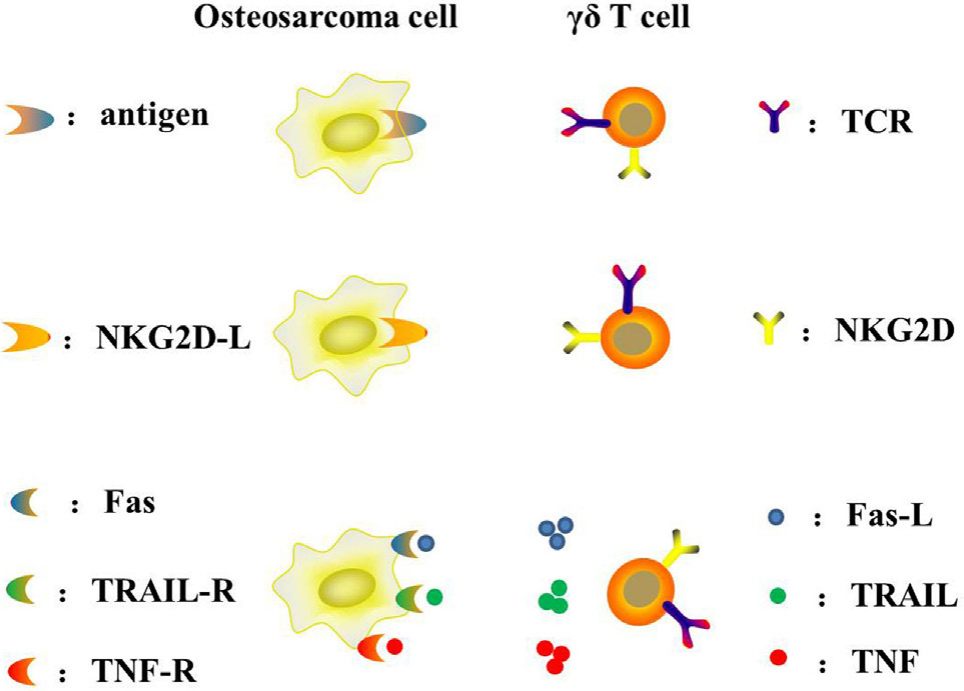
**Mechanism of γδ T cells recognizing and killing osteosarcoma cells**.

γδ T lymphocytes represent a subset of human lymphocytes involved in the innate immune system (Table 2). The peculiar capacity of γδ T cells to directly recognize and lyse osteosarcoma cells was initially documented by Kato et al. (65). The use of adoptive γδ T cell transfer in cancer immunotherapy is a new treatment, especially with regard to osteosarcomas. The main advantages and disadvantages of adoptive γδ T cell transfer immunotherapy are summarized below: (1) it is MHC-independent (all patients may benefit); (2) it is not affected by immune-escape MHC-downregulation; (3) it is not restricted to any precise sarcoma histotype; (4) it has high rates of *ex vivo* expansion with simple protocols; (5) it has limited persistence *in vivo*; and (6) its recognition is limited to extracellular targets (64). Unlike αβ T cells, γδ T cells can naturally recognize tumor antigens in an MHC-independent manner without antigen processing. The particular antigens that γδ T cells recognize are non-peptide, phosphoantigens instead of protein antigens (66). Their recognition and interaction with target cells mostly rely on γδ TCR and other receptors such as NKG2D/NKG2D-L, TRAIL/TRAIL-R, FAS/FAS-L, and TNF/TNF-R (67) (Figure 3). Indirect activity may be mediated through secretion of Th1 and Th2 activating cytokines (68–70).

**Table 2:** **Evaluations of γδ T cell therapy in OS**.

Ancillary therapy	Study type	Comment
None	*In vitro* (65)	Markedly enhanced cytotoxicity against the antigen-pulsed tumor cells as compared with untreated tumor cells
ZA	*In vitro* (75)	Potent antitumor activity and the enhanced immunosensitivity of OS cell lines to γδ T cells
IFN-γ	*In vitro* (22)	Enhancement of susceptibility of tumor cell lines, HOS and U2OS, to the cytotoxicity of γδ T cells
ZA	*In vitro and in vivo* (78)	More efficient ability to inhibit tumor growth and potent antitumor activity
Trastuzumab + ZA	*In vitro* (77)	Enhancement of cytotoxicity of γδ T cells against ZA-sensitized OS cells

Two different immunotherapeutic strategies can be used to harness γδ T cells for cancer immunotherapy: adoptive transfer of *ex vivo* expanded γδ T cells, or combination of γδ T cells and agents, such as aminobisphosphonates (NBPs). Zoledronic acid (ZA), the most potent NBPs, can act on tumor cells by inhibiting tumor cell adhesion to mineralized bone as well as tumor cell invasion and proliferation (71, 72). Moreover, the antitumor effect of NBPs in bone cancer metastases due to prostate cancer, lung cancer, and other solid tumors has been documented, supporting the clinical utility of NBPs in the treatment of bone metastases (73, 74). *In vitro and in vivo* studies show that ZA significantly enhances the killing activity of γδ T lymphocytes against osteosarcoma cells (65, 75, 76). Furthermore, combination of Trastuzumab [an anti-HER-2 monoclonal antibody (mAb)] and Vγ9Vδ2 T cells can enhance the cytotoxicity against ZA-sensitized osteosarcoma cells (77). A central mechanism mediating this activity utilizes the Fas/Fas-L pathway, and it was demonstrated that expression of Fas-L may be modulated in osteosarcoma by IFN-γ. Thus, combination of adoptive transfer of γδ T cells and IFN-γ may enhance anti-osteosarcoma activity and provide a new approach to the therapy of osteosarcoma (22). In the future, the role of γδ T cell immunotherapy combined with other modalities needs to be elucidated.

Clinical trials studying the effects of γδ T cell immunotherapy for both hematological malignancies and solid tumors show promise for cellular therapy of these cancers (78). Although many studies suggest potential use of γδ T cells to treat osteosarcoma, only preclinical studies using osteosarcoma cell lines have been done so far (75, 79). These studies document potential anti-osteosarcoma activity of γδ T cells and justify evaluating γδ T cells in clinical trials of osteosarcoma. Such clinical studies now seem to be technically feasible, as a result of the recent advances in *ex vivo* expansion of γδ T cells (80).

### Gene-Engineered Tumor-Specific T Cells

Genetic engineering of T lymphocytes can endow them with new antitumor specificities, and may facilitate the successful clinical adoption of immunotherapy techniques. Progress in gene-transfer technology has made it possible to impart precise and functionally active TCRs or CARs into conventional T cells (Figure 2). This means that recognition of TSAs may be acquired through inducing the cytomembrane expression of transgene-encoded TCRs or CARs. Here, we will focus on TCR or CAR redirected T cells as possible pivotal treatments for osteosarcoma that may be developed in the near future.

#### TCR-Engineered T Cells

To a great extent, *ex vivo* expansibility of antitumor lymphocytes has so far confined the clinical translation of many adoptive immunotherapy approaches in solid tumors. However, advances in the ability to effectively engineer T lymphocytes may actually address this challenge through introducing tumor-specific TCR genes into T cells (81–83). T cells with transgenic T-cell receptors (tgTCRs) and a matched endogenous CD3 complex can be activated upon encountering their respective antigen presented by HLA molecules and then specifically target tumor cells. The success of adoptive transfer TCR gene-transduced T cells from recent preclinical and clinical studies mainly depends on (1) the expression level of tgTCRs on the cell surface; (2) the intrinsic affinity of the intended tgTCRs; and (3) the differentiation state of the modified T cells (84–87).

This approach, nevertheless, faces many fundamental challenges, including (1) low affinity of the TCR binding its peptide/MHC complex; (2) decrease in TCR expression of the introduced and endogenous TCRs; (3) mispairing of the introduced α/β chains with endogenous α/β TCR chains; and (4) potential risks of autoimmune responses and toxicities. Not only may new encoded α/β chains form undesired dimers with the endogenous TCR chains lowering the expression and the efficacy of the intended antitumor tgTCR, but also can give rise to unintended recognition of different antigens with potential risks of autoimmune responses and toxicities (88, 89). Strategies being explored to address these limits include structural modifications of TCRs (90–92), transduction of γδ T cells or hematopoietic stem cells later differentiated into T cells (93, 94), construction of single-chain antitumor TCRs (95) and silencing the expression of the endogenous TCR (96, 97). On the other hand, some research teams focus on entirely bypassing the MHC restrictions through producing another artificial design, the CAR (38). It is important to note that, relative to TCR affinity, “the more, the better” may be untenable. Artificially induced high affinity of TCR may eventually impair the functions of antitumor T cells and generate side effects (82).

Despite these obstacles, MART-1-, gp100-, or NY-ESO-1-specific TCR T cells have been evaluated in melanoma patients with favorable outcomes (59, 85, 98). Tumor regression in patients with metastatic synovial cell sarcoma can be achieved by using gene-engineered lymphocytes reactive with NY-ESO-1; this study represented the first demonstration of the successful treatment of a non-melanoma tumor using TCR-transduced T cells (59). These observations indicate that TCR-based gene therapies directed against NY-ESO-1 can represent a new and effective therapeutic approach for solid tumors. By coincidence, NY-ESO-1 in osteosarcoma cell line U2OS and HOS can be elevated following demethylating treatment with DAC (21). Although data about the usage of tgTCR T cells in treating osteosarcoma patients is not available, studies of this issue are critical, as suggested by the encouraging results with NY-ESO-1-specific TCR T cells.

#### Chimeric Antigen Receptor-Engineered T Cells

Emerging strategies with CAR-engineered T cells, based on principles of synthetic biology are hypothesis-generating and thought-provoking, and have ushered in what may prove to be major advances in T-cell-based immunotherapy. In general, the CAR consists of three parts: an extracellular antigen recognition domain [a single-chain variable fragment (scFv) from a mAb], a hinge, and an intracellular signaling domain (99). Existing CARs use the CD3ζ chain as the signaling domain and additionally contain other signaling domains involved in T cell activation or costimulation (83). In contrast to the TCR, the scFv domain of CAR binds directly to recognize intact target molecules expressed on cell surfaces in an MHC-independent fashion. This approach can be applied to all patients regardless of their HLA-haplotype. The limitation of CAR-engineered T cells is their inability to target intracellular antigens. CAR-based strategies can bypass the need for MHC-restricted antigen presentation and are, thus, insensitive to tumor escape mechanism related to HLA downmodulation (100). Moreover, CARs also overcome most of the T cell triggering limitations due to low epitope density, since the scFv of the mAb is characteristic of high affinity for the antigen target. For instance, adoptive transfer of HER2-specific T cells can overcome low levels of HER2 expression in osteosarcoma (23).

Chimeric antigen receptor-T therapy was initially investigated for treatment of hematologic malignancies, partly because of deep understanding of the lineage-restricted surface expression of antigens and easy delivery of modified T cells to tumor sites within the blood. CD19 is viewed as one of the most successful antigen targets to date. CD19-CAR-T cell therapy, proven to be feasible and safe, mediates potent anti-leukemic responses both in children and young adults with hematologic cancers (18). Additionally, considerable effort has been spent by several groups to explore the use of CAR-T therapy in preclinical models to treat sarcomas by targeting interleukin-11 receptor α-chain (IL-11Rα), NK receptor ligands (NKG2D-L), and fetal acetylcholine receptors (101–103). Based on these studies, CAR-T therapy has the potential in clinical application *in vivo* both as a primary treatment for sarcoma and as a complementary modality for sarcoma in the future. For instance, human epidermal growth factor receptor 2 (HER2) is expressed by the majority of human osteosarcomas. HER2 could potentially become a prognostic marker and therapeutic target for osteosarcomas (104). Unlike breast cancers, HER2 expression is quite low in osteosarcoma cells and non-engineered T cell therapy cannot effectively target it. However, the adoptive transfer of HER2-specific CAR-T cells can circumvent this limitation and cause regression of osteosarcoma in preclinical models of loco-regional lesions as well as experimental models of pulmonary metastases (23). In clinical trials, adoptive transfer of HER2-CAR-T cells in patients with osteosarcoma indicates that these cells can persist for 6 weeks without evident toxicities. Hence, combination of HER2-CAR-T cells and other immunotherapies should be pursued to promote their expansion and persistence (20).

The CAR-T therapy is actually closely related to immunotherapy based on mAbs. The main advantage of mAbs over CAR-T approach is their convenient storage and easy usage. While CAR-T therapy needs advanced expertise (105). When antigen presentation and TIL burden are low, immunomodulating mAbs may not induce a strong antitumor response. But CAR-T cells are not inhibited by these barriers (106). Furthermore, other therapeutic challenges concerning CAR-T cell include persistence and expansion, trafficking, tumor microenvironment and efficacy in solid tumors (99). Here, we must point out that the usage of CAR-T cells may result in the development of expected or unexpected toxicities. Therefore, selecting a target antigen for engineering a CAR is crucial. For example, if the target antigen is not only expressed on tumors but also on normal tissues, the possibility for on-target toxicities to occur can be predicted. On-target toxicities result from the recognition of an intended molecular target expressed both on tumors and normal tissues, whereas in the case of off-target toxicities, T cells recognize an unintended structure due to antigenic mimicry or cross-reactivity. On-target toxic events were relatively mild. For example, dose-limiting liver toxicity was observed in patients receiving anti-CAIX CAR-T lymphocytes for renal cell carcinoma (107). Besides the relatively manageable events, recent trials have also reported the occurrence of fatal on-target off-tumor toxicities. A severe lung toxicity, cytokine storm and subsequent multi-organ failure occurred shortly after the CAR-engineered T cell infusion, likely due to recognizing of low HER2 expression levels in normal lung cells. It was demonstrated that a marked increase in IFN-γ, GM-CSF, TNF-α, IL-6, and IL-10 occurred shortly after the lymphocyte infusion (108). Incorporation of suicide genes or engineering of multi-specific CAR-T cells to limit their activation to tumor sites might allow control of adverse events (109). In summary, adoptive transfer of CAR-T cells appears to be an attractive therapeutic option for experimental osteosarcoma therapy, but further research will be required to develop comprehensive measures to avoid adverse side effects.

Further studies are needed to identify unique antigens expressed on tumor cells and not in normal tissues. So far, several candidate antigens have been identified that are either aberrantly expressed by tumors (e.g., CTAs, such as MAGE family or NY-ESO-1, in the formation of peptide/HLA complex) or overexpressed in tumors compared with normal tissues (such as HER2) (52, 110, 111). In view of these target antigen candidates, clinical trials with osteosarcoma-specific TCR or CAR-redirected T lymphocytes are planned for the coming years.

## Future Outlook

Theoretically, T cells are capable of eliciting an effective antitumor response and causing significant tumor regression. However, ATCT for osteosarcoma presents unique challenges, including the inherent heterogeneity of the tumor itself, the complexity and importance of the tumor microenvironment, and the limited therapeutic accessibility to tumor site. In order to address these challenges, we will consider the following three issues.

### Promising Molecular Targets

The clinical benefit of T-cell-based immunotherapeutics in the control of a diverse set of human cancers occurs, mainly as a result of the selection of therapeutic targets. Selecting a promising molecular target is required for the successful adoptive transfer T cell therapy. For osteosarcoma, potential therapeutic molecular targets are summarized in Table 3. CTA (MAGE-A family and NY-ESO-1) and HER2 were discussed in detail above. Another antigen target (IL-11Rα), two immunomodulatory targets (PD-L1 and CTLA-4 checkpoint inhibitor) and one target axis (NKG2D-NKG2D-L) should also be considered.

**Table 3:** **Promising molecular targets for OS immunotherapy**.

Molecular target	Target site	Prognostic marker	Antibody immunotherapy	T-cell-based immunotherapeutics
CTA (MAGE-A family and NY-ESO-1)	Tumor cell	Unknown	None	Combining demethylating treatment and CD8+ T cells in OS animal models (21)
PD-L1	Tumor cell	Unknown (but PD-1 is correlated with progression of OS) (44)	Blockade of PD-1/PD-L1 interactions in OS mouse models (114)	None
HER2	Tumor cell	Yes (98)	Trastuzumab (77)	γδ T cells against zoledronate-sensitized OS cells (77); HER2-specific CAR-T therapy in OS mouse models (23)
IL-11Rα	Tumor cell	Unknown	None	IL-11Rα-CAR+ T cells successfully killing human OS cells and inducing the regression of OS with lung metastases (96)
CTLA-4	T cell	Unknown	Combining CTLA-4 blockade and tumor lysate-pulsed DCs or PD-L1 blockade in murine OS (127, 128)	None
NKG2D-L	Tumor cell	Unknown	None	None

Interleukin-11, a member of the Jak–STAT activating family of cytokines, binds to IL-11Rα and transduces the gp130–Jak–STAT signaling pathway, promoting tumorigenesis (112, 113). Increased expression of IL-11Rα occurs in prostate cancer and has been suggested as a candidate target for metastatic prostate cancer lesions (114). Additionally, overexpression of IL-11Rα occurs in both metastatic breast cancer and prostate cancer, implying the involvement of IL-11Rα in bone metastases (114, 115). Additional evidence has demonstrated that IL-11Rα was overexpressed in primary and metastatic osteosarcoma, but not expressed in the adjacent normal lung tissue (102, 116), or in major organs, such as brain, heart, and kidney. Moreover, IL-11Rα-CAR+ T cells successfully killed human osteosarcoma cells and induced the regression of osteosarcoma with lung metastases (102). So it is a possible ligand-directed target in osteosarcoma, and especially for metastatic osteosarcoma. Conceivably, combining IL-11Rα-CAR+ T cells with other therapies may produce a clinically beneficial response in patients with osteosarcoma.

Programed death 1 (PD-1) is a receptor expressed on the surface of T and B lymphocyte subsets, as well as other immune cells. It can mediate T-cell inhibition upon binding with its ligand, which was named programed cell death ligand 1 or B7 homolog 1 (PD-L1 or B7-H1). Recent clinical trials of inhibitory antibodies (aimed at PD-1 or PD-L1) have induced durable tumor regression and continued stabilization of disease in patients with advanced cancers, such as melanoma, renal cell carcinoma, and non-small cell lung cancer (117–119). Currently, three different phase II clinical trials studying the effect of checkpoint inhibitors are ongoing in osteosarcoma patients. One of them is based on anti-PD-1 antibody Pembrolizumab (NCT02301039), and the other two are utilizing anti-PD-1 antibody Nivolumab with or without anti-CTLA-4 antibody Ipilimumab (NCT02304458 and NCT02500797). The results of these three clinical trials will, at least to some extent, elucidate the efficacy of checkpoint inhibitors in patients with osteosarcoma. Evidence reveals that the percentage of PD-1 is significantly upregulated on both peripheral blood CD4+ and CD8+ T lymphocytes from osteosarcoma patients and PD-1 is involved in the progression of osteosarcoma (44). Additionally, high levels of PD-L1 expression both in human osteosarcoma cell lines and tumor samples have also been determined via RNA-based assay for the first time (43). Therefore, inhibition of PD-1/PD-L1 is an interesting therapeutic target that may restore immune system function against osteosarcoma cells. The efficacy of osteosarcoma-reactive CTLs *in vitro* and *in vivo* is significantly enhanced via blockade of PD-1/PD-L1 interactions, resulting in decreased tumor burden and increased survival in the osteosarcoma metastasis models (120). Therefore, the combination of adoptive CD8+ T cell and blockade of PD-1/PD-L1 interactions should be pursued, as it is a promising therapeutic strategy for osteosarcomas. In the tumor microenvironment, IFN-γ can increase efficient antigen processing for MHC-mediated antigen presentation and enhance immune response (121). But the combination of PD-1/PD-L1 blockade and IFN-γ needs to be further explored since IFN-γ may simultaneously upregulate the expression of PD-L1 in peripheral tissues and immune cells and, thus, suppress the immune response (122–124).

CTLA-4 (CD152), expressed on activated T cells, can attenuate the antitumor response by downregulating T-cell activation. However, CTLA-4 also may be expressed on tumors, inducing apoptosis of neoplastic cells (125). Therefore, blockade of the inhibitory effects of CTLA-4 or combination of NY-ESO-1 vaccination with CTLA-4 blockade can enhance antitumor response in metastatic melanoma patients, resulting in clinical benefits (126, 127). It is interesting that long-term survival of patients with advanced melanoma has been achieved by using Tremelimumab, an anti-CTLA-4 antibody (128). Currently, several meta-analyses consistently show that CTLA-4 is significantly associated with osteosarcoma risk, and might play an important role in carcinogenesis of osteosarcoma (129–132). To prevent immune-escape and obtain complete control of a carcinoma, combination immunotherapy of CTLA-4 and PD-L1 blockades was investigated in animal models of metastatic osteosarcoma (133). Combining anti-CTLA-4 antibody and tumor lysate-pulsed DCs can promote antitumor reaction in murine osteosarcomas (134). These data indicate that combination of CTLA-4 blockade with other immunotherapies against osteosarcoma shows great clinical promise.

NKG2D, initially identified on NK cells, is also found to be expressed by CD8+ T cells, NKT cells, and γδ T cells. Its ligands consist of MHC class I-related chains A and B (MICA, MICB) and the UL16-binding protein family (ULBP1-6) (135). Via NKG2D–NKG2D-L axis, both NK cells and T cells can be activated and mediate cytotoxicity (136). It is validated that NK cells can target osteosarcoma cells in an NKG2D–NKG2D-L dependent manner (137). However, there is a paucity of data about T cell targeting of osteosarcomas via this mechanism. A positive correlation between NKG2D-L expression and improved clinical outcomes has been documented in various solid tumors (138). In osteosarcoma patients, a deficiency of MICA–NKG2D-mediated immunesurveillance is revealed by prevalent expression of MICA and higher serum level of soluble MICA (139). These data suggest that sustaining or increasing NKG2D-L expression on osteosarcoma cells or NKG2D on T cells may be a viable strategy for developing effective cancer immunotherapy.

It is widely accepted that TSAs, such as CTA, are ideal target antigens that are only expressed on tumor tissues but not in normal tissues. As compared with TAAs, TSAs cannot stimulate the immune response toward self, inducing, or exacerbating cancer-associated autoimmune diseases, which is fundamentally different for TCR- and CAR-based strategies. Epidermal growth factor receptor vIII mutant (EGFRvIII), a TSA target, is a recurrent oncogenic variant found in 25–64% of glioblastomas (140–142). Genetically modified T cells have been used to target EGFRvIII, only expressed on glioblastoma cells, to establish a basis for future clinical application (141, 143, 144). Although EGFR expression is common in osteosarcoma tumors, EGFRvIII (the most common mutant type of EGFR) (145) is absent from osteosarcoma tumors (146). Osteosarcoma is characteristic of genomic rearrangements and genomic instability. Chromothripsis (tens to hundreds of genomic rearrangements occurring in a one-off cellular crisis) is more likely to occur in osteosarcomas than in other tumor types. Moreover, evidence shows that chromothripsis can drive the development of tumor due to copy number changes and/or dysregulated gene expression (147). Therefore, new potential therapeutic targets may be identified by next-generation sequencing studies (148).

### Tumor Microenvironment

In order to allow for rapid tumor growth, an assortment of non-neoplastic cells is recruited to nurture the expanding neoplasm. These cells are required to support the development of the tumor by synthesizing matrix proteins, cytokines, and fabricating the vascular network needed for nutrition and waste exchange of the neoplastic tissues. The tumor microenvironment influences the protein expression of healthy surrounding tissues and the process of tumorigenicity (149). This provides a possible new paradigm for cancer therapy by targeting the “soil” instead of only the “seed” (150). Improved knowledge of tumor microenvironments involved in tumor progression, invasiveness, and metastasis may improve the efficacy of therapeutic strategies, and ultimately have a significant clinical impact.

Most solid tumors possess a stromal compartment that promotes tumor growth directly through cell contact or paracrine secretion of cytokines, growth factors, and nutrients, thereby influencing tumor-induced immunosuppression (150, 151). During the process of T cell activation, a parallel inhibitory program that will eventually stop the response is also fully activated (152). Immunosuppressive mechanisms at play in the tumor microenvironment involve the suppressive action of regulatory T cells (Tregs), myeloid-derived suppressor cells (MDSCs), tumor-associated macrophages (TAMs), stromal fibroblasts, and no doubt, other cell types not yet defined. These cells inhibit T cell function by upregulating the expression of surface molecules that bind inhibitory receptors, such as CTLA-4, PD-1, TIM-3, LAG-3, and BTLA, as well as through producing immunosuppressive cytokines or other soluble factors (153, 154). Early-phase trials of antibodies that interfere with the T cell checkpoint molecule PD-1 have shown clinical efficacy in diverse tumor types, including melanoma, lung cancer, bladder cancer, stomach cancer, and renal cell cancer (155). Therefore, targeting components of the tumor microenvironment, such as the immunosuppressive cytokines, inhibitory receptors on T cells, tumor vasculature, and cancer-associated fibroblasts, may be an innovative approach to the treatment of osteosarcoma.

### Combination Strategy

Chemotherapy, radiation therapy, vaccines, and immune checkpoint inhibitors, to name a few, partnered with adoptive T-cell transfer and/or components of the tumor niche, may yield meaningful clinical benefits. Rational combinations of immunotherapies are already showing increased efficacy in murine models and human patients (156). Clinical investigations of immune checkpoint inhibitors have demonstrated activity in multiple types of neoplasms (157). So checkpoint inhibitors may be an attractive component of combination strategy for treatment of osteosarcoma in the future (158).

## Conclusion

Based on recent insights into the biology and immunology of osteosarcoma, harnessing the body’s immune system especially via ATCT to enhance treatment of osteosarcoma is becoming an increasingly attractive option. Monotherapy is insufficient to carry a universal cure for tumors and future studies need to focus on identifying the optimal combination strategy of immunotherapy with surgical therapy, chemotherapy, and/or radiation therapy.

## Author Contributions

This review paper was written by ZW, revised by BL and YR, and guided by ZY.

## Conflict of Interest Statement

The authors declare that the research was conducted in the absence of any commercial or financial relationships that could be construed as a potential conflict of interest.
